# Development of a Sensitive Self-Powered Glucose Biosensor Based on an Enzymatic Biofuel Cell

**DOI:** 10.3390/bios11010016

**Published:** 2021-01-07

**Authors:** Kantapat Chansaenpak, Anyanee Kamkaew, Sireerat Lisnund, Pannaporn Prachai, Patipat Ratwirunkit, Thitichaya Jingpho, Vincent Blay, Piyanut Pinyou

**Affiliations:** 1 National Nanotechnology Center, National Science and Technology Development Agency, Thailand Science Park, Pathum Thani 12120, Thailand; kantapat.cha@nanotec.or.th; 2 School of Chemistry, Institute of Science, Suranaree University of Technology, 111 University Avenue, Suranaree, Muang, Nakhon Ratchasima 30000, Thailand; anyanee@sut.ac.th; 3 Department of Applied Chemistry, Faculty of Science and Liberal Arts, Rajamangala University of Technology Isan, 744, Suranarai Rd., Nakhon Ratchasima 30000, Thailand; sireerat.in@rmuti.ac.th; 4 SCiUS, Suranaree University of Technology, 111 University Avenue, Suranaree, Muang, Nakhon Ratchasima 30000, Thailand; pannaporn2003@gmail.com (P.P.); patipatguitar@gmail.com (P.R.); thitichaya.jingpho@gmail.com (T.J.); 5 Division of Biomaterials and Bioengineering, University of California San Francisco, 513 Parnassus Ave, San Francisco, CA 94143, USA

**Keywords:** glucose, self-powered, biosensor, biofuel cell, NAD-glucose dehydrogenase, glucose oxidase, horseradish peroxidase, reduced graphene oxide

## Abstract

Biofuel cells allow for constructing sensors that leverage the specificity of enzymes without the need for an external power source. In this work, we design a self-powered glucose sensor based on a biofuel cell. The redox enzymes glucose dehydrogenase (NAD-GDH), glucose oxidase (GOx), and horseradish peroxidase (HRP) were immobilized as biocatalysts on the electrodes, which were previously engineered using carbon nanostructures, including multi-wall carbon nanotubes (MWCNTs) and reduced graphene oxide (rGO). Additional polymers were also introduced to improve biocatalyst immobilization. The reported design offers three main advantages: (i) by using glucose as the substrate for the both anode and cathode, a more compact and robust design is enabled, (ii) the system operates under air-saturating conditions, with no need for gas purge, and (iii) the combination of carbon nanostructures and a multi-enzyme cascade maximizes the sensitivity of the biosensor. Our design allows the reliable detection of glucose in the range of 0.1–7.0 mM, which is perfectly suited for common biofluids and industrial food samples.

## 1. Introduction

Bioenergetics is a source of inspiration for researchers in different fields. Known living organisms obtain energy from metabolism and electron transport chain reactions, in which adenosine triphosphate (ATP) is produced. Many enzymes and redox cofactors are involved in the electron transport chain in a highly regulated manner, and the process is highly efficient (ca. 80% efficiency) [1,2]. The idea of exploiting or harvesting energy from biological fluids containing organic substances, such as carbohydrates or lactate, and an oxidant like oxygen, using redox biocatalysts led to the invention of biofuel cells (BFCs) [3]. A typical biofuel cell consists of a bioanode, in which the fuel is oxidized by a biocatalyst (namely microbes or enzymes) immobilized on the conductive surface of an electrode, and a biocathode, which contains biocatalysts capable of reducing an oxidant like O_2_ or H_2_O_2_ [4]. When the bioanode and biocathode are assembled, electrical power can be produced. Although the power output generated from current BFCs is considerably lower than that from conventional fuel cells [5], BFCs offer advantages for sensors in clinical analysis. In particular, physiological conditions and mild temperatures are suitable for BFCs, whereas fuel cells normally operate under severe conditions and high temperatures. In addition, biofuel cells can be miniaturized and adapted to medical applications, including implanted power systems [6], tattoo sensors [7,8], and wearable biosensors [9].

Most electrochemical (bio)sensors use an external potentiostat to adjust the potential of the working electrode and to measure the current extracted from the analyte. By contrast, one advantage of self-powered biosensors is that no external power supply or potentiostat is needed [10,11]. In self-powered biosensors, the power output and voltage difference depend on the rates of the reactions at the biocathode and bioanode and, particularly, on the analyte concentration in the BFC, thus enabling sensing [12]. This concept of utilizing a BFC for sensing was first demonstrated by Katz et al. [13]. Besides, self-powered enzymatic sensors can be designed to operate based on an inhibition mechanism, with higher concentrations of enzyme inhibitor lowering the power output [14]. Self-powered sensors based on biofuel cells are compatible with common readout systems, such as digital multimeters [15], electrochromic displays [16,17], and LEDs [18], thus reducing costs and facilitating portability for point of care applications [19]. 

The determination of glucose concentration is crucial to some applications in the food industry [20] and in the clinical analysis of blood and other biofluids [21]. For example, over 460 million people live with diabetes today, requiring regular monitoring of glucose levels (in the case of type I diabetes, 4–10 daily measurements can be recommended) [22]. Glucose–O_2_ biofuel cells have been used to build self-powered glucose sensors, using glucose-oxidizing enzymes on the bioanode combined with oxygen-reducing enzymes on the biocathode [23,24,25,26]. A selection of such systems is compiled in Table S1. In addition, self-powered glucose sensors have also been proposed with non-enzymatic components (abiotic BFCs) [27,28].

**Figure 1:**
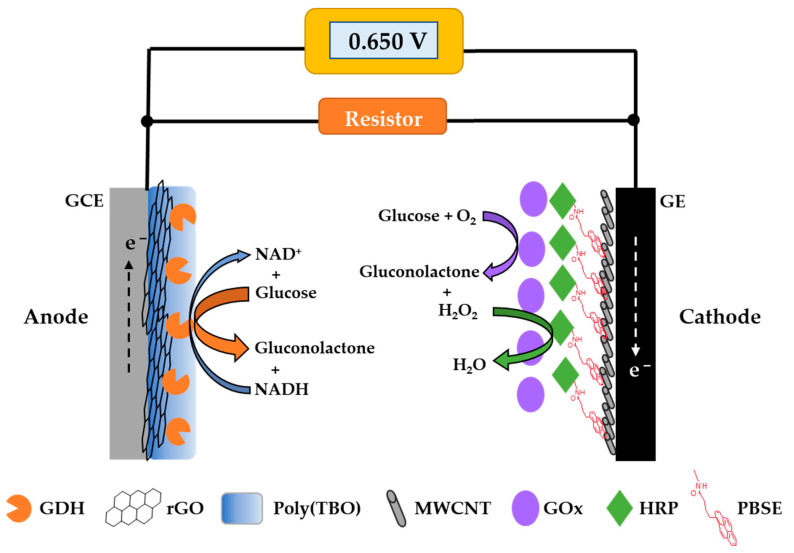
Scheme of the glucose self-powered biosensor developed in this work based on an enzymatic biofuel cell. GDH: glucose dehydrogenase, rGO: reduced graphene oxide, Poly(TBO): poly(toluidine-blue O), MWCNT: multi-wall carbon nanotube, GOx: glucose oxidase, HRP: horseradish peroxidase, PBSE: 1-pyrenebutyric acid *N*-hydroxysuccinimide ester, GCE: glassy carbon electrode, GE: graphite electrode.

In this work, a glucose BFC was developed and investigated for its potential and performance as a glucose self-powered biosensor (Figure 1). Optimizing the electron transfer between the redox enzymes and the electrodes is essential to achieve the desired sensing performance [29]. The bioanode designed was based on nicotinamide adenine dinucleotide-dependent glucose dehydrogenase (NAD-GDH) modified with poly(toluidine-blue O) (poly(TBO)), used for NAD^+^ regeneration, and reduced graphene oxide (rGO) on the surface of a glassy carbon electrode. For the biocathode, a multi-enzyme system based on glucose oxidase (GOx) and horseradish peroxidase (HRP) was employed, avoiding the buildup of hydrogen peroxide, a product of glucose oxidation by GOx. Both enzymes were co-immobilized onto multi-wall carbon nanotubes (MWCNTs) to improve the electrical contact between HRP and the graphite electrode surface, aiming to increase the potential from the cathode. Both GDH and GOx use the same substrate, glucose, and thus this BFC can be operated as a single-compartment cell. The resulting BFC was investigated for the detection of glucose in different samples with excellent results.

## 2. Materials and Methods

### 2.1. Chemical and Materials

*D*-glucose, toluidine blue O (TBO), glutaraldehyde, and dimethylformamide (DMF) were obtained from TCI Chemicals (Japan). 1-pyrenebutyric acid *N*-hydroxysuccinimide ester (PBSE), glucose dehydrogenase (GDH) from *Pseudomonas* sp., and β-Nicotinamide adenine dinucleotide hydrate from yeast were obtained from Sigma-Aldrich (St. Louis, MO, USA). Glucose oxidase (GOx) from *Aspergillus niger* and peroxidase from horseradish (HRP) were obtained from TCI Chemicals (Japan). The enzyme solutions used for electrode preparation were prepared to attain the following concentration: GDH 5 mg/mL, GOx 3 mg/mL, and HRP 1 mg/mL. MWCNTs and graphene oxide nanocolloids (nano-GO) were purchased from Sigma-Aldrich (St. Louis, MO, USA). The MWCNTs used had a size of O.D. × L = 6–13 nm × 2.5–20 μm, with a purity of >98%. The nano-GO was dispersed in water (2 mg/mL) prior to use. All aqueous solutions were prepared with deionized (DI) water.

### 2.2. Preparation of Electrodes

The bioanode was prepared by modifying a glassy carbon electrode (GCE, 3 mm) purchased from CH Instruments (Austin, TX, USA). Prior to modification, the GCE was polished on a polishing micro-cloth with a sequence of alumina powders of 1, 0.3, and 0.5 microns. Then the GCE was rinsed with DI water and sonicated in ethanol for 2 min to remove any alumina particles left. rGO was deposited on the GCE surface by electrochemical reduction of graphene oxide (GO) using a recent protocol [30]. Briefly, 4 μL of 0.5 mg/mL GO in water was drop-casted on the surface of the GCE and allowed to dry under an infrared lamp for 20 min. The GO-modified GCE was subsequently transformed into rGO by cycling the electrode in 0.1 M citrate phosphate buffer at pH 6.5 across a potential range of −1.5 to 1.5 V (vs. Ag/AgCl 3 M KCl) at a scan rate of 100 mV/s for 20 cycles and then it was rinsed with DI water.

Next, poly(TBO) was electropolymerized on the rGO/GCE electrode surface, adapting a procedure from [31]. In particular, rGO/GCE was immersed in a solution containing 1 mM TBO and 0.1 M HCl and the electrode was swept from −0.2 to 0.6 V (vs. Ag/AgCl 3 M KCl) at a scan rate of 100 mV/s for 30 cycles. The modified electrode was rinsed with DI water and dried in air before adding 2 μL of GDH solution to the electrode surface. After the electrode was dried at room temperature, the electrode was crosslinked by exposing it to vapor from a solution of 5% glutaraldehyde in water at 4 °C overnight.

The biocathode was prepared from a graphite electrode (GE, 6 mm). The GE was insulated by using Teflon film and was polished with emery paper prior to modification. A 5 mg/mL MWCNT suspension in DMF was dispersed by sonication for 20 min and 15 μL of the suspension were drop-casted on the GE surface. The obtained MWCNT/GE was incubated with 10 μL of 10 mM PBSE in DMF for 1 h before thoroughly rinsing it with DMF and phosphate buffer, respectively. Then 16 μL of HRP were drop-casted on the GE surface. When the HRP layer was dry, 10 μL of GOx were added to the HRP layer to coat the electrode surface. It was then allowed to dry and kept at 4 °C in the fridge. The anode and cathode were rinsed with phosphate buffer at pH 7.4 before any measurement.

### 2.3. Electrochemical Measurements

An Emstat Blue plus 3 potentiostat (Palmsens, the Netherlands) was employed to perform the electrochemical measurements. A three-electrode setup was used for voltammetric and amperometric experiments, consisting of a Pt wire as an auxiliary electrode, a Ag/AgCl 3 M KCl reference electrode (Italsens, Italy), and the modified bioanode/biocathode as the working electrode. The catalytic current due to glucose oxidation on the bioanode was evaluated by chronoamperometry at a constant applied potential of 0.2 V (vs. Ag/AgCl 3 M KCl). An OWON B35 digital multimeter (Zhangzhou, China) was used for reading out the self-powered detection of glucose.

### 2.4. Biofuel Cell and Self-Powered Glucose Measurement

The performance of the biofuel cell was evaluated by assembling the bioanode and the biocathode to a two-electrode electrochemical system in a single-compartment electrochemical cell containing 40 mM glucose and 5 mM NAD^+^ in 0.1 M phosphate buffer at pH 7.4 under air-saturated conditions. The bioanode was connected to the working electrode cable while the biocathode was connected to the combined counter/reference electrode cables. The anode and cathode were immersed in a buffer containing glucose until the open-circuit voltage (OCV) was attained. Then, a multiple-potential chronoamperometry with successive potential steps ranging from 0.010 to 0.65 V against the OCV of the BFC was performed. The solution was continuously stirred at 500 rpm using a magnetic stirring bar. The volume for the self-powered detection was 8 mL and the distance between anode and cathode was 5 mm. The current response was recorded at each potential to obtain the polarization and power curves. The self-powered biosensor for glucose detection was performed by assembling the anode and the cathode with the multimeter and connecting it to an external resistor (24 kΩ unless indicated otherwise) in parallel (Figure 1). The current generated passing through the resistor was calculated using Ohm’s law.

## 3. Results and Discussion

### 3.1. Electrochemical Characterization

#### 3.1.1. Bioanode Characterization and Optimization

NAD-GDH is the enzyme immobilized on the anode to oxidize glucose to gluconolactone, as shown in Equation (1).

*D*-glucose + NAD^+^ → *D*-gluconolactone + NADH + H^+^ (1)

Compared to other redox enzymes, such as pyrroloquinoline quinone (PQQ)-GDH and flavin adenine dinucleotide (FAD)-GDH, NAD-GDH has the advantage that it is specific towards glucose, whereas PQQ-GDH and FAD-GDH display a broader substrate specificity [32]. Moreover, catalysis by GDH enzymes is insensitive to oxygen. NAD^+^ is a necessary cofactor, as it is the primary electron acceptor for NAD-GDH. Thus, it has to be supplied for measurements using this enzyme [33]. One important consideration when using NAD-dependent enzymes is the need to regenerate NAD^+^ from NADH. Direct oxidation of NADH can be achieved if a sufficiently high overpotential is applied to the electrode [34], but this tends to cause undesirable electrode fouling [35]. Phenothiazines (e.g., toluidine blue and methylene blue) and phenoxazines (Nile blue and cresyl violet) have been widely used for the regeneration of NAD^+^ at lower potentials [34,36,37]. In this work, we chose toluidine blue O (TBO), a redox dye based on phenothiazines, to facilitate the oxidation of NADH.

Prior to modifying the poly(TBO) on the electrode surface, we incorporated reduced graphene oxide onto the bioanode to increase its specific surface area and improve the electrical conductivity between the enzyme and the electrode surface [38]. The electrochemical reduction of GO through cyclic voltammetry was chosen to control the extent of reduction of the GO. We measured the IR absorption spectra of the starting GO and the resulting rGO (Figure S1), which confirmed that the number of oxygen-containing functional groups, such as C–O (1060 cm^−1^), C–OH (1226 cm^−1^), and C=O (1733 cm^−1^) [39], decreased as the GO is electrochemically reduced. Moreover, we checked the electrode active surface area of the GO- and rGO-modified electrode using a standard solution of 5 mM Fe(CN)_6_^3+^ containing 0.1 M KCl. The results also indicated that the electrode active surface area of the rGO-modified electrode increased significantly when GO was partially converted to rGO (Figure S2). After introducing rGO on the glassy carbon electrode, the poly(TBO) film was deposited using cyclic voltammetry in TBO acidic solution.

**Figure 2:**
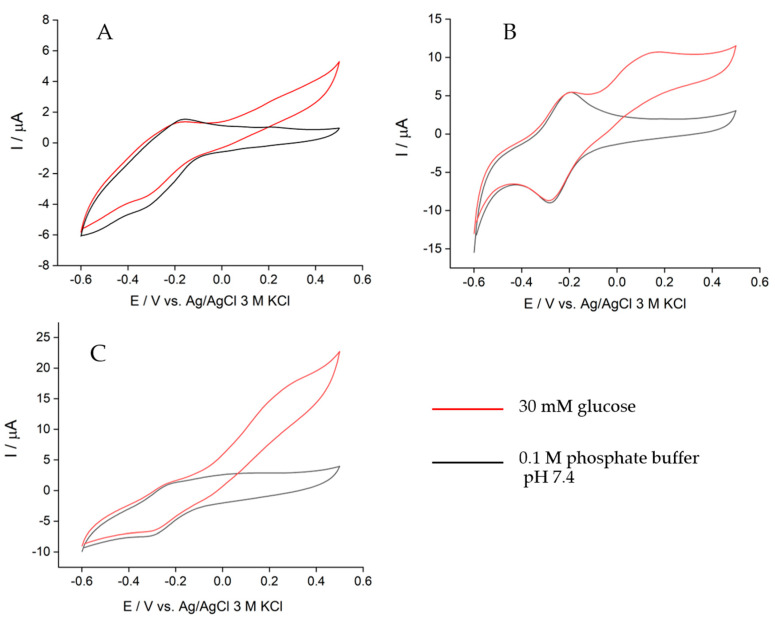
Cyclic voltammograms of the bioanode with different modifications: (**A**) GDH/poly(TBO)/GCE, (**B**) GDH/poly(TBO)/GO/GCE, and (**C**) GDH/poly(TBO)/rGO/GCE in the presence of 0.1 M phosphate buffer pH 7.4 containing 5 mM NAD^+^ and 30 mM glucose (red) and the absence of glucose (black) at a scan rate of 10 mV/s, air-saturating conditions.

The effect of the electrode modifications on the catalytic current delivered by the bioanode was examined using cyclic voltammetry. In Figure 2A, GDH was co-immobilized with poly(TBO) on the GCE surface. A catalytic current was observed upon glucose addition (red line), with an onset potential of ca. 0 V. The catalytic current obtained with this design was the lowest of all electrodes prepared due to its limited surface area, which led to small amounts of poly(TBO) and GDH attached to the electrode. The cyclic voltammogram of the bioanode modified with GO is depicted in Figure 2B, showing an onset potential of ca. −0.1 V in glucose oxidation. A pronounced anodic peak is observed at 0.17 V, which corresponds to the oxidation of NADH facilitated by poly(TBO). Thus, introducing GO on the electrode significantly increases the catalytic current compared to the original GCE. Furthermore, in the absence of glucose (black line), the GDH/poly(TBO)/GO/GCE design exhibited a pronounced peak due to the TBO reversible redox couple, with a midpoint potential of ca. −0.24 V, in agreement with previous reports [40]. This is due to the abundance of oxygenated functional groups in GO. These groups may assist in the adsorption of TBO during electropolymerization, but they may also be reduced during operation. TBO is positively charged and may thus adsorb on the GO via electrostatic and other intermolecular interactions [41,42].

The cyclic voltammogram for GDH/poly(TBO)/rGO/GCE is presented in Figure 2C. The highest catalytic current was observed for this configuration, with GO being partially reduced. By increasing the rGO/GO ratio, the electroactive surface area increased, which is favorable to the reaction rate. However, the decrease in the number of oxygenated functional groups may decrease TBO adsorption on the electrode surface and make the formation of the poly(TBO) film difficult [41,42]. The rGO-modified bioanode showed an onset potential of ca. −0.1 V, similar to that for the GO bioanode, and an anodic peak at 0.35 V due to the oxidation of NADH. The increase in current at potentials above 0.4 V is due to the direct oxidation of NADH without mediation by poly(TBO). Based on these results, the rGO-modified bioanode design was selected for further optimization.

**Figure 3:**
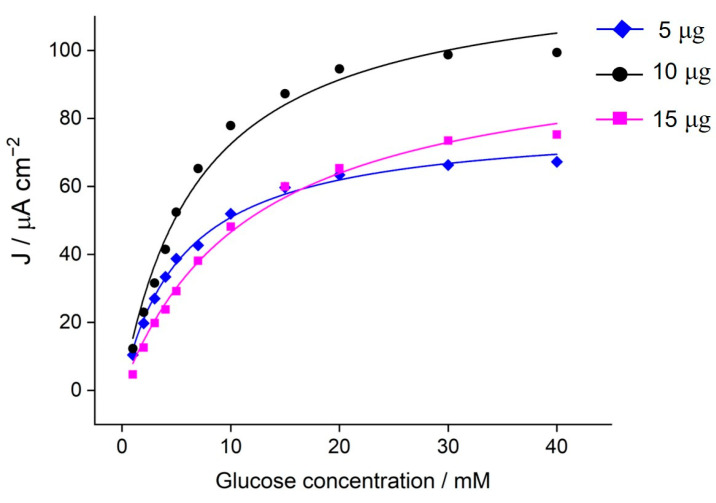
Effect of GDH load on the poly(TBO)/rGO/GCE on the catalytic current. Chronoamperometry was performed in the presence of 5 mM NAD^+^ in 0.1 M phosphate buffer pH 7.4 at the applied potential of 0.2 V vs. Ag/AgCl 3 M KCl. Solid curves correspond to Michaelis–Menten fits (see Table S2 for details).

The effect of the GDH amount immobilized on the poly(TBO)/rGO/GCE design is shown in Figure 3. To ensure a fast electron transfer rate, the load of enzyme should be adjusted based on the rest of redox components. In this case, the amounts of the other redox components, rGO and poly(TBO), were fixed, and the amount of GDH was varied from 5 to 15 μg. Chronoamperometry was used to evaluate the catalytic current of the bioanode at an applied potential of 0.2 V. The catalytic current increased significantly when the enzyme load increased from 5 to 10 μg (Figure 3). However, when the GDH amount was further increased to 15 μg, the catalytic current decreased notably. The results for the different enzyme loadings approximately follow Michaelis–Menten kinetics (Equation (2)):

(2) $$J=\;\frac{J_{max}\left[Glucose\right]}{K_{m}+\left[Glucose\right]}$$

where *J_max_* and *K_m_* are adjustable parameters of the fit. The apparent *K_m_* continuously increased with the enzyme load (Table S2), suggesting the possibility of diffusional limitations. A thick film of GDH may hinder the diffusion of glucose and the electron transfer, thus lowering the catalytic current. Still, the catalytic efficiency (*J_max_*/*K_m_*) was maximal for an intermediate loading of 10 µg. From these results, an enzyme load of 10 μg was chosen for subsequent experiments. For this enzyme load, a maximum current density of ca. 100 μA cm^−2^ was observed under saturating glucose conditions, with an apparent *K_m_* value of 8.63 mM (Table S2).

#### 3.1.2. Biocathode Characterization

To develop a single-compartment BFC as a glucose self-powered sensor, the reactions on the anode and cathode should not interfere with each other. Thus, for the biocathode, we chose GOx and HRP biocatalysts, which are capable of catalyzing glucose oxidation, and to further reduce the H_2_O_2_ converted into non-interfering H_2_O (Figure 1). Bi-enzymatic biosensors based on GOx and HRP have been widely investigated, especially for amperometric biosensors [43]. Although using GOx as a biocatalyst in the BFC is advantageous in terms of high specificity for glucose, the performance of this enzyme can be affected by the oxygen level. Another potential disadvantage of GOx is the production of H_2_O_2_, which in the long term can deteriorate the enzyme itself. Thus, the reduction of H_2_O_2_ by HRP can also favor the long-term stability of the BFC. Thanks to the small size of HRP, a direct electron transfer between the enzyme and the electrode can take place efficiently [44]. Moreover, HRP possesses a relatively high potential for H_2_O_2_ reduction: when HRP is supported on a (nanostructured) carbon-based electrode, this value can exceed 0.6 V [45,46]. Maximizing the cathode potential directly increases the potential difference between anode and cathode and, thus, the power output of the BFC [46]. This is highly desirable as it can increase the sensitivity of the sensor.

**Figure 4:**
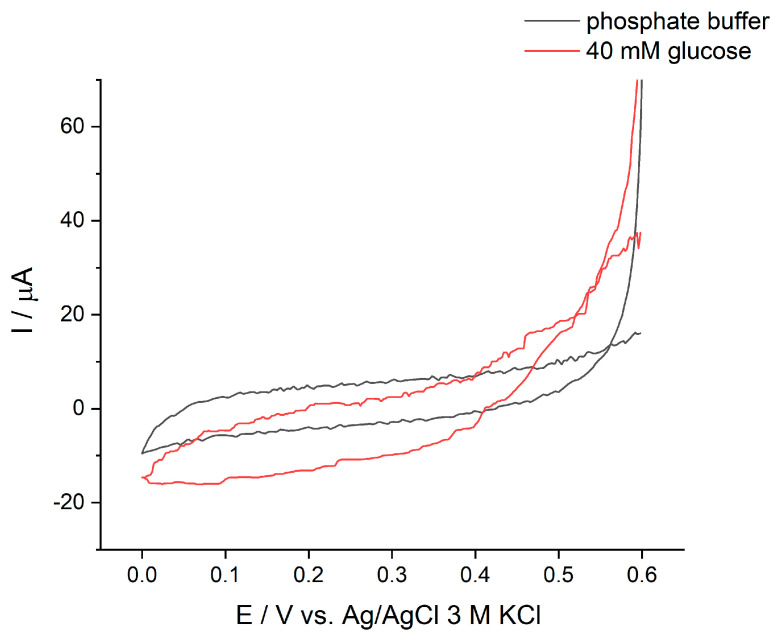
Cyclic voltammograms of the GOx/HRP/MWCNT/GE biocathode in the absence and presence of 40 mM glucose in 0.1 M phosphate buffer pH 7.4 at a scan rate of 1 mV/s, air-saturating conditions.

For the biocathode, we incorporated MWCNTs on the graphite electrode (GE) in order to increase the surface area and enable a larger enzyme loading than that on the bare GE. To strengthen the immobilization of HRP on MWCNT, PBSE was used to modify this enzyme. This molecule reacts covalently with amino groups on the enzyme and decorates it with pyrene moieties, which have high affinity for the walls of MWCNTs through π-π stacking interactions [47]. GOx was directly immobilized by adsorption onto the layer of HRP. The electron transfer pathway of the proposed biocathode is presented in Figure 1. Cyclic voltammetry was used to characterize the HRP/GOx biocathode in the absence and presence of glucose, as shown in Figure 4. When 40 mM glucose was added, the reduction current was observed, with an onset potential around 0.53 V, corresponding to the reduction of H_2_O_2_ catalyzed by HRP.

### 3.2. Biofuel Cell Performance

**Figure 5:**
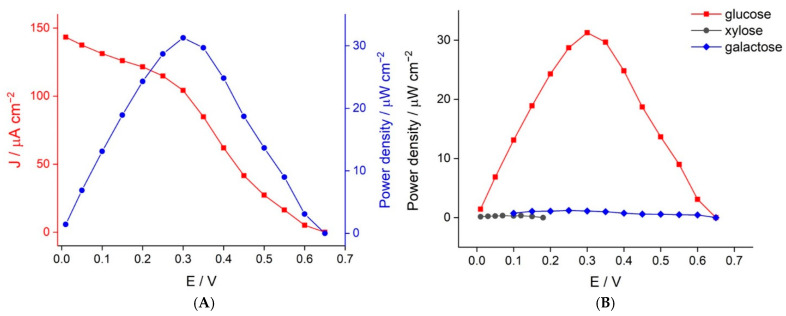
(**A**) Polarization and power density curves of the enzymatic biofuel cell operated in 40 mM glucose. (**B**) Power density curves of the enzymatic BFC in different sugars at a concentration of 40 mM in 0.1 M phosphate buffer pH 7.4 containing 5 mM NAD^+^.

The performance of the glucose BFC was evaluated by assembling the GDH/poly(TBO)/GO/GCE bioanode and the GOx/HRP/MWCNT/GE biocathode as a cell. Both the bioanode and biocathode catalyze the conversion of the same substrate, thus allowing a membrane-less configuration in which both electrodes are in close proximity. This is beneficial to minimize the ohmic loss of the BFC [48]. Moreover, our proposed BFC can be operated under ambient conditions and does not require an extra supply of oxygen to the cathode as an oxidant. The polarization and power densities of the proposed BFC are shown in Figure 5A. The OCV of the BFC was 0.65 V with a maximum power density of 31.3 μW cm^−2^ at a potential of 0.3 V in the presence of 40 mM glucose. The resulting OCV obtained agreed well with the individual open-circuit potentials (OCPs) of the bioanode (−0.12 V) and the biocathode (0.55 V) at 40 mM glucose. The obtained power output clearly shows that the ohmic drop was low, as it matched very well to the expected theoretical power output (0.55 + 0.12 = 0.67 V).

For the designed BFC-based sensor, we anticipated that sugars other than glucose could be the main interferents present in clinical samples. Thus, we also evaluated the power output of the BFC when provided with different sugars. The results are shown in Figure 5B. The OCV values observed when operated with 40 mM xylose and galactose were 0.18 and 0.65 V, respectively. Although the OCV output from galactose is the same as that obtained from glucose, the current output (and therefore the power output) was very low. Therefore, the designed BFC-based sensor showed excellent selectivity towards glucose.

### 3.3. Self-Powered Glucose Detection

The proposed BFC was investigated as a self-powered glucose sensor. To this end, the BFC was connected to the resistor and the multimeter, as shown in Figure 1. As mentioned above, no external power or potential was required to generate the signal, and a multimeter served as the readout system. We explored the use of three different resistors (5 kΩ, 10 kΩ, and 24 kΩ). The results, shown in Figure S3, suggest that the resistance might be used to modulate the sensitivity and dynamic range of the sensor. For use with submicromolar glucose concentrations, a low resistance may be preferred, which may increase the change in current density at low glucose concentration. For use with glucose concentrations in the range expected for biofluids (<7 mM), we found that 10 kΩ is a reasonable setting (Figure S3).

**Figure 6:**
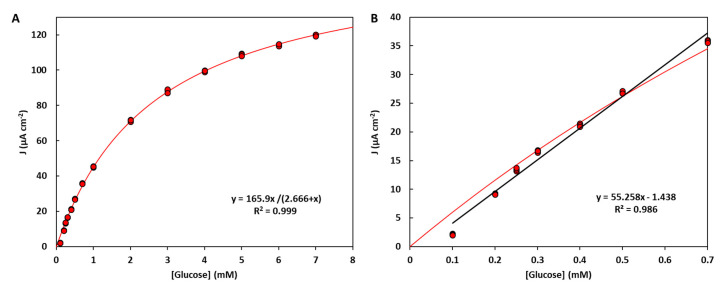
Calibration curve for the self-powered detection of glucose (*n* = 3) in a membrane-less cell configuration, using the optimized electrodes and a 10 kΩ resistor setting. The calibration curve can be well described using a Michaelis–Menten law and used across a broad glucose concentration range (**A**), with parameters *J_max_* = (165.9 ± 1.6) µA cm^−2^, *K_m_* = (2.67 ± 0.06) mM. A conventional linear calibration can also be used for selected ranges, such as the low concentrations shown in panel (**B**).

At low glucose levels, current densities showed a linear dependence with glucose concentration. A linear range for glucose determination was obtained for the concentration range of 0.1–0.70 mM, with a notable sensitivity of 55.3 μA cm^−2^ mM^−1^ (Figure 6B). It should be noted here that the linear range for the self-powered glucose differs from that for the bioanode on its own (Figure 3) because here we have the contribution from the bioanode and the biocathode, which are run in a self-powered mode. Furthermore, the proposed sensor can be used to determine glucose concentration across a broader dynamic range. To this end, we can use Equation (2) as a nonlinear calibration curve, as shown in Figure 6A. To determine glucose concentration, we can simply solve the calibration curve, as shown in Equation (3). This strategy allows us to determine glucose concentration in the range of 0.1–7 mM, which is perfectly suited to a variety of clinical needs.

(3) $$\left[Glucose\right]=\;\frac{K_{m}}{J_{max}-J}$$

Finally, we performed glucose determination based on this set-up for a sample containing glucose. The measurement was validated by spiking glucose into a sample solution (a 0.2 mM glucose drink formula for diabetic patients) and checking the recovery percentage. The percent recovery was 109 ± 5% when spiking 0.10 mM glucose in the sample solution, measured from three replicates. Unlike BFCs for power systems, high power output may not be imperative for sensing applications. Compared to previous designs (Table S1), the reported self-powered glucose sensor exhibits good sensitivity and high OCV, and a dynamic range well suited for the detection of glucose at the single-digit micromolar and submicromolar levels typical of common biofluids.

## 4. Conclusions

This work demonstrates a new design of an enzymatic biofuel cell that can be used as a self-powered glucose sensor. The redox enzymes NAD-GDH and GOx/HRP were incorporated on the electrode surfaces as biocatalysts. Moreover, carbon materials with high specific surface area (rGO and MWCNTs) were employed to maximize the current density and improve the electrical contact between the enzymes and the electrode surface. Compared to previous systems, the sensor can be operated as a single-compartment cell without a membrane between the anode and cathode. Conveniently, the sensor can be operated under air-saturated electrolyte conditions and benefits from the excellent selectivity towards glucose characteristic of GOx. The use of an enzymatic cascade on the cathode allows for boosting the potential of the cell (OCV = 0.65 V), and therefore the sensitivity of the sensor. The dependence of the current density on glucose concentration showed an excellent sensitivity. By using a nonlinear calibration, the detector could determine concentrations in the range of 0.1–7.0 mM. Such a measurement range seems well suited to determine glucose concentration in common biofluids, such as blood (4–7 mM) and urine (<0.8 mM). Samples with higher concentrations can be conveniently diluted and buffered for use with this system. Given its promise for clinical analysis, we are currently working on miniaturizing the system and validating its use with biofluids against established methods.

## Data Availability

Not applicable.

## Supplementary Materials

The following are available online at https://www.mdpi.com/2079-6374/11/1/16/s1, Table S1: comparison of self-powered glucose sensors, Figure S1: IR absorption spectra of GO and rGO, Figure S2: cyclic voltammograms of GO and rGO with ferricyanide, Table S2: Michaelis-Menten parameters at different enzyme loadings, Figure S3: optimization of the external resistor.
