# Supplementary material for: Development of a Sensitive Self-Powered Glucose Biosensor Based on an Enzymatic Biofuel Cell
Source: Biosensors (Basel). 2021 Jan 7;11(1):16. doi: 10.3390/bios11010016 (PMC7825672; doi:10.3390/bios11010016)
Supplement: Supplementary file 1 [file biosensors-11-00016-s001.pdf]

biosensors

MDPI

SUPPORTING INFORMATION

Development of a Sensitive Self-Powered Glucose Biosensor based on an Enzymatic Biofuel Cell

Kantapat Chansaenpak [1], Anyanee Kamkaew [2], Sireerat Lisnund [3], Pannaporn Prachai [4], Patipat Ratwirunkit [4], Thitichaya Jingpho [4], Vincent Blay [5,*] and Piyanut Pinyou [2,*]

[1] National Nanotechnology Center, National Science and Technology Development Agency, Thailand Science Park, Pathum Thani 12120, Thailand.
[2] School of Chemistry, Institute of Science, Suranaree University of Technology, 111 University Avenue, Suranaree, Muang, Nakhon Ratchasima 30000, Thailand.
[3] Department of Applied Chemistry, Faculty of Science and Liberal Arts, Rajamangala University of Technology Isan, 744, Suranarai Rd., Nakhon Ratchasima 30000, Thailand.
[4] SCiUS, Suranaree University of Technology, 111 University Avenue, Suranaree, Muang, Nakhon Ratchasima 30000, Thailand.
[5] Division of Biomaterials and Bioengineering, University of California San Francisco, 513 Parnassus Ave, San Francisco, CA 94143, USA.

* Correspondence: piyanutp@sut.ac.th (P.P.), vincent.blayroger@ucsf.edu (V.B.)

Table S1. Comparison of the performance of previously developed enzymatic biofuel cells or hybrid enzymatic biofuel cells for self-powered glucose sensing.

Electrode	Catalyst (Anode/Cathode)	Fuel	Oxidant	Operating conditions	P_{max} ($\mu W\ cm^{-2}$)	OCV (V)	Dynamic range (mM)	Ref.
Anode: Pt-Ir wire<br>Cathode : carbon cloth	NAD-GDH/BOD	Glucose	O_2	0.1 M phosphate buffer<br>pH 7.0, 10 mM glucose<br>Air-saturated buffer	11.8	0.43	1 - 10	[1]
Anode: TTF-CF<br>Cathode: CF<br>(microneedle electrodes)	GOx/Pt-Rh alloy	Glucose	O_2	0.1 M phosphate buffer<br>pH 7.4, 80 mM glucose<br>Air-saturated buffer	170	0.38	10 - 80	[2]
Anode: TTF-PCI<br>Cathode: PCI<br>(screen printed electrode array)	GOx/BOD	Glucose	O_2	1 M phosphate buffer<br>pH 7 , 100 mM glucose<br>Air-saturated buffer	120	0.57	1 - 25	[3]
Anode: MWCNTs-ink<br>Cathode: Ag_2O/Ag<br>(stretchable textile-based electrodes)	GOx/-	Glucose	-	0.1 M PBS buffer pH 7,<br>50 mM glucose	160	0.44	0 - 50	[4]
Anode: CoPc/PBA/Buckypaper<br>Cathode: PBA/Buckypaper	GOx/MnO_2	Glucose	-	0.1 M PBS buffer pH 7,<br>20 mM glucose	136	0.65	0.5 - 8	[5]
PBSE/Buckypaper based on MWCNTs	PQQ-GDH/laccase	Glucose	O_2	0.1 M phosphate buffer<br>pH 6.0, 45 mM glucose<br>Air-saturated buffer	67.86	0.682	0.5 - 35	[6]
Anode: rGO/poly(TBO) Cathode :<br>PBSE/MWCNTs	NAD-GDH/<br>GOx-HRP	Glucose	H_2O_2	0.1 M phosphate buffer<br>pH 7.4, 40 mM glucose<br>Air-saturated buffer	31.3	0.65	0.1 – 7.0	This work

CF = carbon fibers; TTF = tetrathiafulvalene; PCI Porous carbon ink; GOx = glucose oxidase; GDH = glucose dehydrogenase; HRP = horseradish peroxidase; BOD = bilirubin oxidase

TBO = toluidine blue; MWCNTs = multi-walled carbon nanotubes; CoPc = cobalt phthalocyanine; PBA = pyrenebutyric acid; PBS = Phosphate buffered saline;

PBSE = 1-pyrenebutyric acid *N*-hydroxysuccinimide ester.

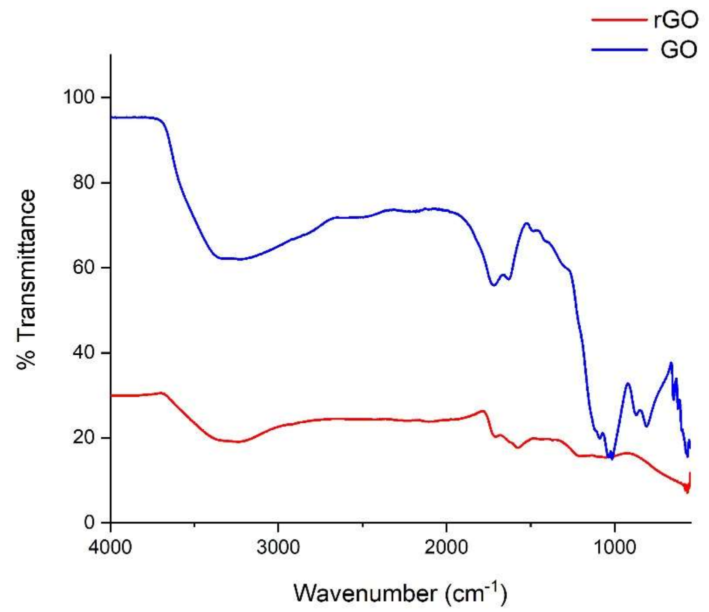

Figure S1. IR spectra for rGO and GO.

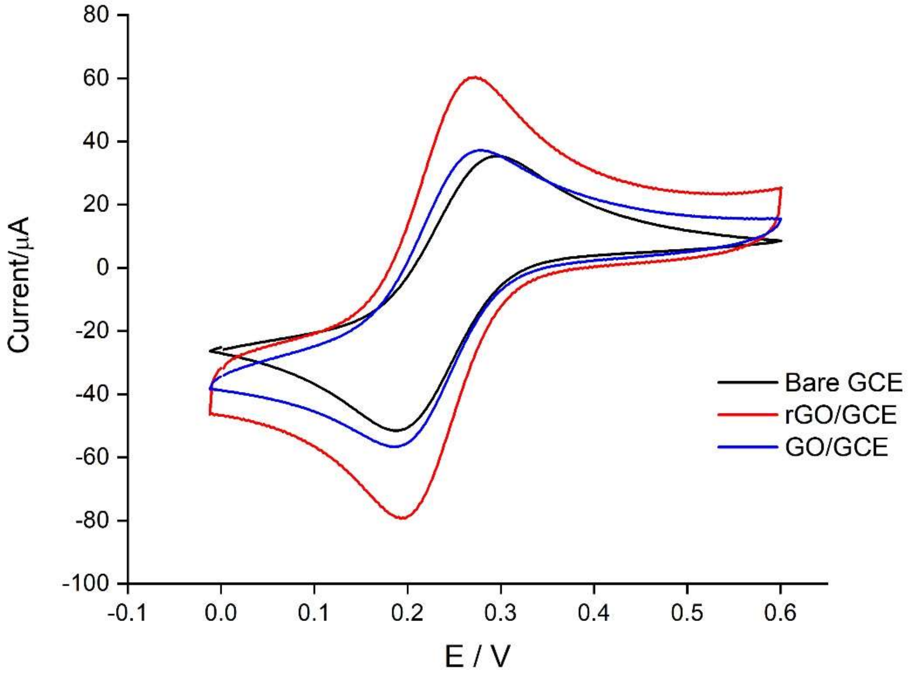

Figure S2. Cyclic voltammograms of the modified electrodes in the solution of 5 mM $Fe(CN)_6^{3+}$ containing 0.1 M KCl at the scan rate of 50 mV/s.

Table S2. Comparison of the Michaelis-Menten parameters, including K_m and J_{max}, for the results in Figure 3.

GDH amount (µg)	K_m (mM)	J_{max} (µA cm^{-2})	J_{max}/ K_m (µA mM^{-1} cm^{-2})
5	5.6	79.1	14.1
10	7.1	123.7	17.4
15	11.9	101.9	8.6

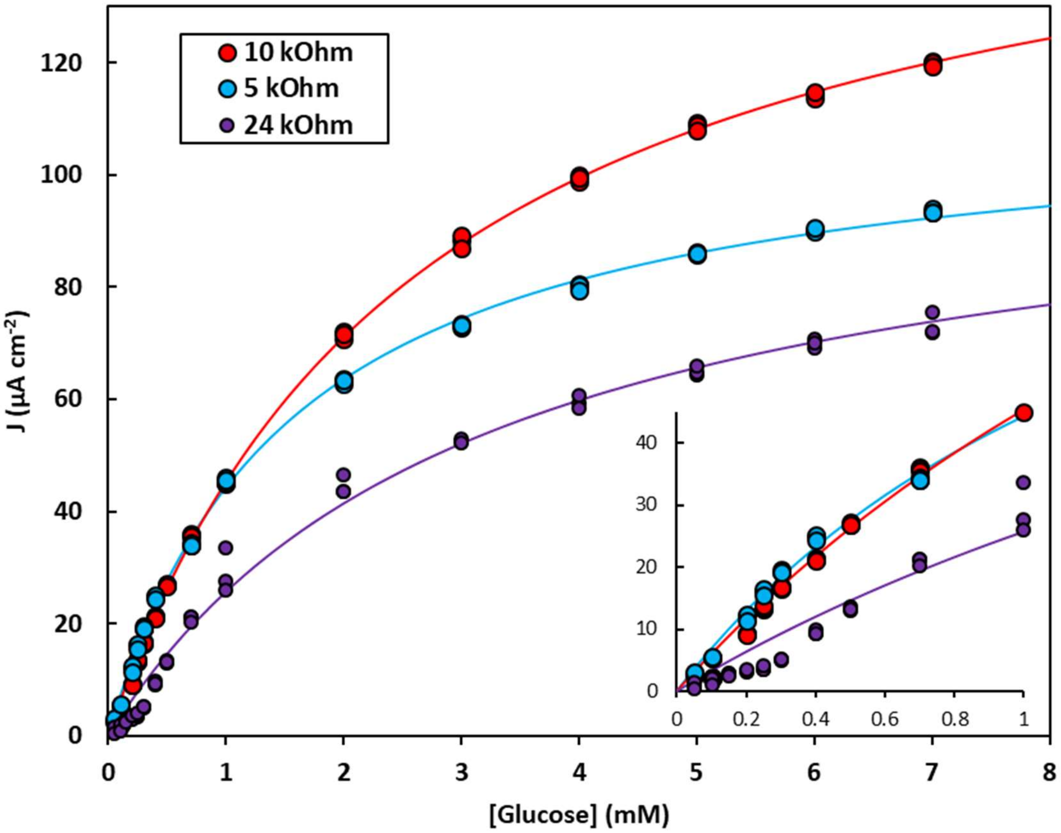

Resistance (kΩ)	K_m (mM)	J_{max} (µA cm^{-2})	R^2
5	1.547±0.030	112.8±0.8	0.9989
10	2.666±0.059	165.9±1.6	0.9991
24	3.191±0.225	107.5±3.4	0.9951

Figure S3. Calibration curve for the self-powered detection of glucose (n = 3) in a membrane-less cell configuration, using the optimized electrodes and different resistor settings. The calibration curve can be well described using a Michaelis-Menten law and used across a broad concentration range (A), with the parameters indicated in the table.

References

1. Huang, S.-H.; Chen, W.-H.; Lin, Y.-C. A Self-Powered Glucose Biosensor Operated Underwater to Monitor Physiological Status of Free-Swimming Fish. Energies 2019, 12, 1827.
2. Valdés-Ramírez, G.; Li, Y.-C.; Kim, J.; Jia, W.; Bandodkar, A.J.; Nuñez-Flores, R.; Miller, P.R.; Wu, S.-Y.; Narayan, R.; Windmiller, J.R., et al. Microneedle-based self-powered glucose sensor. Electrochemistry Communications 2014, 47, 58-62, doi:10.1016/j.elecom.2014.07.014.
3. Shitanda, I.; Fujimura, Y.; Nohara, S.; Hoshi, Y.; Itagaki, M.; Tsujimura, S. Paper-Based Disk-Type Self-Powered Glucose Biosensor Based on Screen-Printed Biofuel Cell Array. Journal of The Electrochemical Society 2019, 166, B1063-B1068, doi:10.1149/2.1501912jes.
4. Jeerapan, I.; Sempionatto, J.R.; Pavinatto, A.; You, J.-M.; Wang, J. Stretchable biofuel cells as wearable textile-based self-powered sensors. Journal of Materials Chemistry A 2016, 4, 18342-18353, doi:10.1039/C6TA08358G.
5. Hao, S.; Zhang, H.; Sun, X.; Zhai, J.; Dong, S. A mediator-free self-powered glucose biosensor based on a hybrid glucose/MnO2 enzymatic biofuel cell. Nano Research 2021, 14, 707-714, doi:10.1007/s12274-020-3101-5.
6. Slaughter, G.; Kulkarni, T. A self-powered glucose biosensing system. Biosensors and Bioelectronics 2016, 78, 45-50, doi:10.1016/j.bios.2015.11.022.
